# p.L1795F LRRK2 variant is a common cause of Parkinson’s disease in Central Europe

**DOI:** 10.21203/rs.3.rs-4378197/v1

**Published:** 2024-05-29

**Authors:** Miriam Ostrozovicova, Gertrud Tamas, Petr Dušek, Milan Grofik, Vladimir Han, Petr Holly, Robert Jech, Katarina Kalinova, Peter Klivenyi, Norbert Kovacs, Kristina Kulcsarova, Egon Kurca, Alexandra Lackova, Hamin Lee, Patrick Lewis, Veronika Magocova, Maria Marekova, David Murphy, Jan Necpal, David Pinter, Miroslava Rabajdova, Evžen Růžička, Tereza Serranova, Katarzyna Smilowska, Krisztina Soos, Igor Straka, Tatiana Svorenova, Peter Valkovic, Katerina Zarubova, Zuzana Gdovinova, Henry Houlden, Mie Rizig, Matei Skorvanek

**Affiliations:** Pavol Jozef Safarik University and University Hospital of L. Pasteur and UCL Queen Square Institute of Neurology; Semmelweis University; Department of Neurology and Centre of Clinical Neuroscience, First Faculty of Medicine, Charles University and General University Hospital, Prague, Czech Republic; Jessenius Faculty of Medicine, Comenius University and University Hospital Martin; P.J. Safarik University and University Hospital of L. Pasteur; First Faculty of Medicine, Charles University and General University Hospital in Prague; Charles University in Prague; Gottfried Schatz Research Center, Medical University of Graz; University of Szeged; Medical School University of Pecs; P.J. Safarik University, Kosice; Comenius University and University Hospital Martin; P.J. Safarik University and University Hospital of L. Pasteur; UCL Queen Square Institute of Neurology; Royal Veterinary College; P.J. Safarik University and University Hospital of L. Pasteur; Faculty of Medicine, P. J. Šafárik University; UCL Queen Square Institute of Neurology; Zvolen Hospital; University of Pecs Medical School; Faculty of Medicine, P. J. Šafárik University; Charles University in Prague; First Faculty of Medicine, Charles University and General University Hospital in Prague; Radboud University Medical Centre; Donders institute for Brain, Cognition and Behaviour, Department of Neurology, Parkinson Centre Nijmegen (ParC) Nijmegen; Semmelweis University; Comenius University in Bratislava Faculty of Medicine, University Hospital Bratislava; P.J. Safarik University and University Hospital of L. Pasteur; Comenius University in Bratislava Faculty of Medicine, University Hospital Bratislava and Centre of Experimental Medicine, Slovak Academy of Sciences; Second Faculty of Medicine, Charles University and Motol University Hospital; P.J. Safarik University and University Hospital of L. Pasteur; UCL Queen Square Institute of Neurology and The National Hospital for Neurology and Neurosurgery; UCL Queen Square Institute of Neurology, University College London, Queen Square, London WC1N 3BG, UK.; Slovakia University

**Keywords:** LRRK2, L1795F, Parkinson’s disease, Risk factor, Mutation, Genetics

## Abstract

Pathogenic variants in *LRRK2* are one of the most common genetic risk factors for Parkinson’s disease (PD). Recently, the lesser-known p.L1795F variant was proposed as a strong genetic risk factor for PD, however, further families are currently lacking in literature. A multicentre young onset and familial PD cohort (n = 220) from 9 movement disorder centres across Central Europe within the CEGEMOD consortium was screened for rare *LRRK2* variants using whole exome sequencing data. We identified 4 PD cases with heterozygous p.L1795F variant. All 4 cases were characterised by akinetic-rigid PD phenotype with early onset of severe motor fluctuations, 2 receiving LCIG therapy and 2 implanted with STN DBS; all 4 cases showed unsatisfactory effect of advanced therapies on motor fluctuations. Our data also suggest that p.L1795F may represent the most common currently known pathogenic LRRK2 variant in Central Europe compared to the more studied p.G2019S, being present in 1.81% of PD cases within the Central European cohort and 3.23% of familial PD cases. Together with the ongoing clinical trials for LRRK2 inhibitors, this finding emphasises the urgent need for more ethnic diversity in PD genetic research.

## Introduction

Twenty years ago, the leucine-rich repeat kinase 2 (*LRRK2*) gene was identified as a key driver of Parkinson’s disease (PD) pathophysiology [^1,2^]. Since then, it has received recognition as one of the most common genetic causes of autosomal dominant PD, as well as one of the strongest genetic risk factors in sporadic PD with promising potential for gene-specific disease-modifying therapy [^3^].

**Figure 1:**
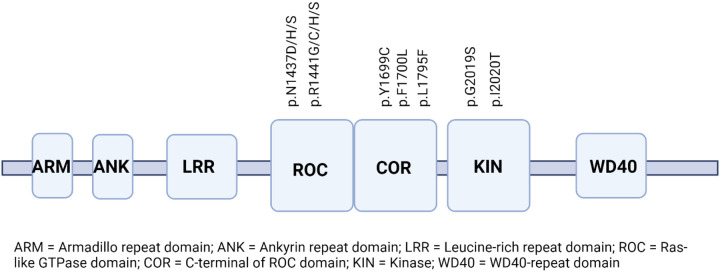
Schematic representation of the *LRRK2* gene with highlighted PD-associated pathogenic mutations including the proposed p.L1795F variant.

The 51-exon *LRRK2* gene encodes a multidomain enzyme of the same name, containing a leucine-rich repeat (LRR) domain, followed by Ras-like GTPase domain (ROC), C-terminal of ROC (COR) domain and kinase (KIN) domain, that represent the LRRK2’s catalytic core [^4^]. Additional domains described are an armadillo repeat domain (ARM), an ankyrin repeat domain (ANK) in the N-terminal non-catalytic region and a WD40-repeat at the C-terminal region [^5^]. Several missense mutations within the catalytic core have been well-characterised as pathogenic for PD, such as p.G2019S and p.I2020T located in the kinase domain, p.N1437D/H/S and p.R1441G/C/H/S located in the ROC domain, p.Y1699C or p.F1700L in the COR domain [^6,7,8^]. The functional consequence of these mutations is a dysregulation of LRRK2 enzymatic activity, including increased phosphorylation of a subset of Rab proteins [^4^]. (Fig. 1). Additionally, several variants were shown to moderately increase the risk for PD, such as p.Q416* and p.A419V located in the ARM domain [^9,10^], p.R1628P and p.M1646T within the COR domain [^10,11,12^] and p.G2385R within the WD40 domain [^13^] Interestingly, a non-coding *LRRK2* variant c.622C > T was nominated by Genome-Wide Association Studies (GWAS) to also affect the LRRK2 expression and mediate PD risk [^14^].

Lately, the p.L1795F variant (rs111910483) was proposed as a strong genetic risk factor for PD, with an estimated OR of 2.5 [^15^]. It was also previously shown to have a functional effect via enhanced kinase activity, providing more evidence for its pathogenicity [^7^]. The p.L1795F variant was previously identified in 2 siblings with PD, within a family with several family members affected in each generation. However, no segregation was shown due to the unavailability of additional family members [^16^], and further reports are lacking in the literature.

The aim of this study was to screen a multicentre early onset and familial PD cohort from 9 movement disorder centres across Central Europe within the Central European Group on Genetics of Movement Disorders (CEGEMOD) consortium [^17^] for rare *LRRK2* variants using whole exome sequencing (WES) data to access the possible contribution of proposed p.L1795F LRRK2 variant to PD in Central Europe.

## Results

The mean age of included PD patients from Central Europe (n = 220) was 53.5 ± 12.9 years of whom 136 (61.8%) were men. Family history was positive in 93 patients (42.3%) and 117 patients (53.2%) developed PD before the age of 40 years. The basic characteristics of the patients included are summarized in Suppl. Table 1.

### Identification of the LRRK2 p.L1795F variant

**Figure 2:**
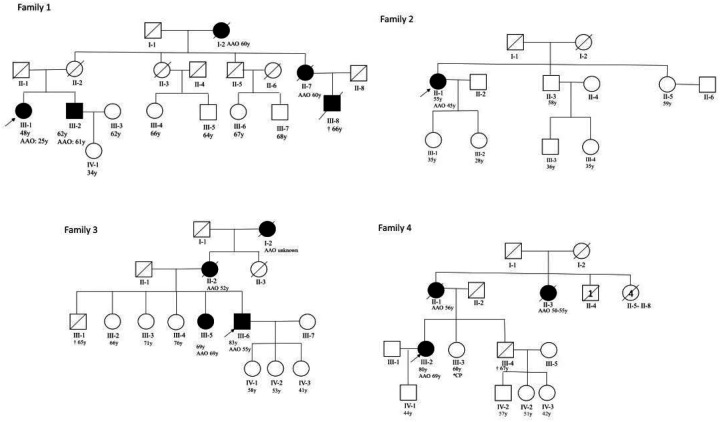
Pedigrees of *LRRK2* p.L1795F positive PD patients. AAO: age at onset; y = years; CP = cerebral palsy;

Leveraging WES data, we identified 3 cases with heterozygous *LRRK2* p.L1795F variant and 1 PD case was additionally identified through clinical genetic testing. (allele frequency = 0.00909; 1.81% within the whole PD cohort and 3.23% of familial PD cases). The age at onset (AAO) was 25 (P1), 45 (P2), 55 (P3) and 69 (P4), respectively (mean AAO = 48.5 ± 18.5), and 3 cases (P1, P3 and P4) had a positive family history (75%) with several family members affected (defined by at least 2 additional affected first-degree, second-degree, or third-degree relatives) showing an autosomal dominant mode of transmission. Pedigrees for all 4 index cases are provided in Fig. 2. Most families report additional members with PD; however, the majority of these individuals were deceased, and biological material was not available to test if the identified *LRRK2* variant segregated with the disease in these cases. Interestingly, all patients were from the same region close to the east Slovak-Hungarian border, with 1 patient (P1) of Hungarian origin and 3 patients (P2–4) from Slovakia (Table 1). In all 4 cases, the p.L1795F variant was validated by Sanger sequencing (Suppl. Figure 1).

### Genotype-phenotype correlation

The clinical features of PD patients harbouring the heterozygous p.L1795F variant are shown in Table 2 and Suppl. Table 2. All 4 cases were characterised as akinetic-rigid PD subtype, with their disease onset as unilateral bradykinesia and rigidity, responsive to levodopa treatment. None of them developed any kind of tremor over the years. Postural instability later developed in all 4 cases (100%), with freezing being present in 2 cases (50%). All 4 cases were characterised by early onset of severe dyskinesia and motor fluctuations, with 2 receiving Levodopa-Carbidopa Intestinal Gel (LCIG) therapy and 2 implanted with Deep Brain Stimulation of the subthalamic nucleus (STN DBS); all these cases showed an unsatisfactory effect of advanced therapies on motor fluctuations.

The MoCA score was normal in 2 patients (50%), 1 patient scored in the PD-mild cognitive impairment range (25%) and 1 in the PD-dementia range (25%), with a mean MoCA score of 22.8 ± 7 and mean PD-CRS total score being 87.7 ± 23.2 (29 ± 1 for cortical and 58.7 ± 22.3 for subcortical score respectively). 3 patients (75%) self-reported neuropsychiatric features, such as depression, anxiety or apathy over the years due to the severity of the illness. The mean score for BDI-II was 23 ± 8.7, PAS was 10 ± 10.1, for PDQ-39 it was 29.9 ± 17.7, and the mean Apathy scale score was 14.8 ± 5.2. For QUIP-RS the mean score was 2.3 ± 4.

Furthermore, patients self-reported additional non-motor features such as urinary urgency or nocturia (100%), light-headedness on standing (100%), chronic pain (75%), insomnia (50%), constipation (50%) or excessive sweating (25%). The mean NMSS score was 48 ± 26.7, the mean SCOPA-AUT score was 16 ± 8.3. The mean ESS score was 5.5 ± 4.5 and for the PDSS-2 it was 23 ± 9.7. RBDSQ score was 3.5 ± 2, with two subjects scoring borderline for REM sleep behaviour disorder (RBD) on RBDSQ (5pts). Nevertheless, polysomnography investigations were not performed in any of these cases. Beta-glucocerebrosidase (GCase) activity measured in 2 cases showed a normal range.

### Structure and function of the p.L1795F variant.

Previous biochemical analysis of the p.L1795F variant reported an approximately five-fold increase in phosphorylation of Rab10 at p.T73, a well-characterised substrate for LRRK2, compared to the wild-type protein, as well as an approximately two-fold increase in autophosphorylation at p.S1292, and a halving of phosphorylation at p.S935 [^7^]. These data are similar to the functional impact of pathogenic mutations in the ROC and COR domain, including p.N1437H, p.R1441C, and p.Y1699C. Notably, the p.L1795F residue is located in close proximity to pathogenic mutations in the ROC and COR domain (Fig. 4). Taken together these data are consistent with, but do not demonstrate, a pathogenic role for the p.L1795F variant.

## Discussion

In this study, we screened for the p.L1795F LRRK2 variant in a multicentre early onset and familial PD cohort within the CEGEMOD consortium [^17^] to determine its possible contribution to PD in Central Europe. In contrast to other European populations, the genetic background of PD in Central Europe remains mostly unknown as genetic reports are scarce. Leveraging WES data, we identified 3 heterozygous PD carriers and 1 heterozygous PD carrier was additionally identified through standard genetic testing. The minor allele frequency (MAF) in our PD cohort was 0.00909, compared to the MAF of 0.0004084 reported previously within the largest PD-associated rare variant meta-analysis so far [^15^]. Similarly, the p.L1795F prevalence in Central European PD patients seems to be much higher compared to the other already-known *LRRK2* pathogenic variants, which are quite rare in Central Europe [^20^]. The p.L1795F variant was present in 1.81% of Central European PD cases and 3.23% of familial PD cases, compared to the prevalence of the most common p.G2019S, which was estimated to be around 0.33% as reported previously [^20^].

Population heterogeneity seems to be a prominent factor in the *LRRK2* allelic distribution. The variants’ prevalence varies geographically, suggesting the locations of founder events and the dispersion of founders’ descendants. The most-studied mutation in *LRRK2*, p.G2019S, was demonstrated to arise independently at least twice in humans [^21^], with one of the founding mutational events occurring in the Middle East approximately 4000 years ago [^16,21,22,23^], leading to its higher frequencies in North African Imazighen (39% of sporadic and 36% of familial PD cases) [^24^], Moroccans (28% of sporadic and 76% of familial PD cases with AD inheritance) [^25^] and Ashkenazi Jewish (13.3% of sporadic and 29.7% of familial PD cases) [^26^], compared to an estimated prevalence of approximately 1% in sporadic and 4% in familial PD patients of European ancestry [^27^]. Similarly, the p.R1441C/G/H is associated with an increased risk of PD in Europeans, especially Hispanics, and West Asians [^28^]. The p.R1441G mutation seems to originate in a Basque population [^29^], being responsible for almost 50% of cases of familial PD cases in that region with high penetrance [^30^]. The pathogenic p.N1437H variant occurs mostly in Scandinavians [^31,32^], while p.N1437S in German [^33^] and p.N1437D in the Chinese population [^34,35,36^]. Additionally, several rare *LRRK2* mutations were identified only in unique families. The p.I2020T represents a rare mutation described in a Japanese family with a single-founder effect [^37,38^], the p.Y1699C was identified in a German-Canadian and British family [^1,2^], the p.I1122V was linked to PD only in one family so far [^1^]. The risk factors p.A419V, p.R1628P and p.G2385R are associated with increased risk in the East Asian PD population [^11,28^], while p.M1646T represents a common mild-risk variant in Europeans [^39^]. In comparison, previous studies in Central Europe showed that *LRRK2* pathogenic variants are quite rare within PD cohorts, with the p.G2019S variant’s combined prevalence being reported as only 0.33% (MAF = 0.00164) [^20^]. The studied p.R1441H, p.N1437H, p.Y1699C, p.I2020T, p. M1869V, p.P755L, p.I1122V, p.L1114=, p. G2385R and p.R1428P were not identified at all. Interestingly, the risk factor p.A419V, which was linked to PD in East and Central Asian populations, was also identified in the Hungarian PD cohort [^20^] and one Polish PD family was reported carrying the p.N1437H variant that is mostly described in Scandinavians [^40^].

The LRRK2 protein possesses two distinct enzymatic activities via its kinase and ROC domains, the second being intimately linked to the COR domain. Interestingly, throughout evolution ROC domains are always accompanied by a COR domain [^41^], therefore they are considered functionally inseparable and described as a ROC-COR supradomain. The disease mechanism underlying *LRRK2*-associated PD is proposed via increased kinase activity with pathogenic mutations leading to gain-of-function and hyperphosphorylation of substrate proteins [^5^]. The GTPase activity has received less attention than the kinase, however, several pathogenic mutations located within the ROC-COR domains indicate that the LRRK2 GTPase activity also plays a role in PD pathophysiology. [^42,43^]. Variants associated within the ROC-COR supradomain were described to reduce the GTPase activity, which in turn elevates the kinase activity [^5,31^,^,44^]. Accordingly, the proposed p.L1795F *LRRK2* variant is located in close proximity to pathogenic mutations in the ROC and COR domain. It has been previously *in vitro* demonstrated to enhance the kinase activity and computationally predicted as likely pathogenic or damaging (*AlphaMissense*: Noted as likely pathogenic with a score of 0.744 [^45^], *REVEL score* = 0.638; *Conservation Score* = 9) [^7^].

*LRRK2*-associated PD is characterised by features consistent with idiopathic PD [^27^]. Initial motor features typically include slowly progressive asymmetric tremor at rest and/or bradykinesia, rigidity and gait abnormalities. The range of disease onset is typically in the 50s and 60s but varies, even within the same family, as early onset (in the 20s) and late onset (in the 90s) have also been described [^46^]. Disease progression also varies significantly among individuals and is related to age of onset [^25,47^]. Nonmotor symptoms (NMS) may appear prior to movement disorder or emerge with motor disease progression, such as hyposmia/anosmia, constipation, depression, anxiety and other neuropsychiatric features [^48^]. Previous studies suggest that sleep disturbances and urinary dysfunction are among the commonly reported NMS by *LRRK2-PD* patients and asymptomatic carriers [^49^]. Certain NMS, especially REM sleep behaviour disorder and cognitive decline, may occur at slightly reduced frequencies compared to sporadic PD [^50,51^].

Different *LRRK2* variants also appear to be linked with specific clinical phenotypes. For instance, the p.R1441G PD carriers may be more likely to develop excessive tremor [^2^]. Motor fluctuations were more likely to develop in p.R1441G/C/H/S compared to the individuals carrying the p.G2019S variant [^52^]. Similarly, the p.G2385R carriers tend to have a more rapidly progressive parkinsonism and motor decline with more motor fluctuations compared to p.G2019S [^53,54^]. Atypical features, such as dementia and amyotrophy were described in individuals carrying the p.Y1699C variant [^1^].

The novel p.L1795F is consistent with the *LRRK2*-associated PD phenotype described in the literature, although it is associated with more rapidly progressive parkinsonism and earlier onset of severe motor fluctuations. The age of onset varies from early (25y) to late (69y) age, similarly as in the first family reported with 2 siblings diagnosed as late onset PD (at 60y and 66y respectively) [^16^]. Interestingly, the patient with significantly younger onset at 25y also carried rare heterozygous *MAPT* p.R538P (c.1613G > C; p.Arg538Pro) variant whose clinical significance is currently unknown (*CADD score* 25.1*; Polyphen*: probably damaging; *SIFT*: deleterious, *carol*: deleterious) as it lacks reports in the literature. Several *MAPT* loci variants have already been reported to interact with the *LRRK2* gene [^55^] and might increase the susceptibility to PD, such as *MAPT* IVS1 + 124 C > G variant that seems to modify the PD risk in *LRRK2* p.G2385R carriers in East Asians, as well as slightly decrease the age of onset [^56^]. Similarly, several polymorphic variations in the *MAPT* gene have already been shown to possibly influence the age of onset in *LRRK2*-associated PD [^57,58^], though the results are inconclusive and the exact mechanism remains unknown. Family history resembled an autosomal-dominant mode of inheritance in 3 cases (75%) as suggested previously [^16^], though additional family members affected were not available for segregation analysis. A striking feature of this variant in all identified cases in our study seems to be the lack of tremor in clinical presentation, as well as very early onset of severe dyskinesia and motor fluctuations, with a narrow therapeutic window and unsatisfactory response to advanced treatment options such as LCIG therapy or DBS. Similarly, in the family carrying p.L1795F variant reported previously, severe dyskinesia was present in 1 PD carrier, though resting tremor was also present at PD onset in 1 sibling contrary to our cohort [^16^]. All 4 patients reported urinary dysfunction and orthostatic hypotension as the most common NMS. Sleep disturbances and constipation were also present in 50% of the carriers identified. None of the p.L1795F carriers were diagnosed with RBD and only one patient was diagnosed with level 1 PD-dementia based on MoCA score (13pts), being in his early eighties and after 28 years of diagnosis. Neuropsychiatric features, such as anxiety or depression were also self-reported in 3 cases (75%). The GCase activity in 2 p.L1795F positive PD cases available showed a normal range, in contrast with the p.G2019S [^59^] or p.M1646T variant [^12^], which are associated with elevated GCase activity in peripheral blood.

## Conclusion

In conclusion, the genetic analysis presented herein, taken together with functional and structural data, supports a pathogenic role for the p.L1795F variant. Therefore, we propose that *LRRK2* p.L1795F variant should be included in the standard genetic testing in PD patients from Central Europe as it seems to represent one of the major contributors to autosomal-dominant PD in this region. It was previously shown to have a high OR and have a functional effect via enhanced kinase activity providing more evidence for its pathogenicity. Our data suggests that p.L1795F may represent the most common currently known pathogenic *LRRK2* variant in Central Europe compared to more studied p.G2019S and therefore should be prioritized. Together with the ongoing clinical trials for *LRRK2* inhibitors, this finding emphasizes the urgent need for more ethnic diversity in PD genetic research.

## Methods

### Study design and participants

We screened Whole Exome Sequencing (WES) data of 219 early onset (below the age of 50 years) or familial PD patients (EOPD = 179, FPD = 93; males 136, mean age = 53.5 ± 12.9 years, mean age of onset = 41.7 ± 11.2 years) recruited from 9 movement disorder centres in Czech Republic, Hungary, Poland and Slovakia within the CEGEMOD consortium as described elsewhere [^17^]. The research protocol was approved by the ethics committees from all participating centres. All patients provided informed consent. Each individual with PD was diagnosed in accordance with the Movement Disorder Society (MDS) clinical diagnostic criteria [^60^]. Demographic and clinical data were collected using standardised protocol. The Movement Disorder Society-Unified Parkinson’s Disease Rating (MDS-UPDRS) Part III [^61^] and Hoehn and Yahr (H&Y) stage systems [^62^] were used to evaluate motor severity. Cognitive functions were evaluated using the Montreal Cognitive Assessment (MoCA) [^63^] and the Parkinson’s Disease - Cognitive Rating Scale (PD-CRS) [^64^]. Non-Motor Symptoms Scale (NMSS) [^65^] was used for the assessment of non-motor symptoms in PD and autonomic dysfunction was reported using the Scale for Outcomes in Parkinson’s disease for Autonomic symptoms (SCOPA-AUT) [^66^]. The REM Sleep Behaviour Disorder Screening Questionnaire (RBDSQ) [^67^] was used as a self-screening tool for RBD. The general level of daytime sleepiness using The Epworth Sleepiness Scale (ESS) [^68^]. Fatigue was measured with the Multidimensional Fatigue Inventory (MFI) [^69^]. The Beck Depression Inventory II (BDI-II) [^70^] and the Parkinson Anxiety Scale (PAS) [^71^] were used to assess for mood problems. The Impulsive-Compulsive Disorders were reported via the Questionnaire for Impulsive-Compulsive Disorders in Parkinson’s Disease-Rating Scale (QUIP-RS) [^72^]. The 39-Item Parkinson’s Disease Questionnaire (PDQ-39) was used to assess PD-specific health related quality over the last month [^73^].

### Genetic Studies

Peripheral blood was obtained from all individuals and genomic DNA was extracted from 0.5ml of frozen EDTA whole blood samples using QIAamp DNA Blood Mini Kit (Qiagen, UK), based on the manufacturer’s instructions, after obtained informed consent from all participants. Samples were then quantified and checked for purity using a NanoDrop spectrophotometer (Thermo Scientific) and whole exome sequencing (WES) was performed by Novogene (Cambridge, UK) according to their protocol [^74^]. The libraries were sequenced with Illumina’s NovaSeq 6000. All samples passed the internal QC steps with an average of 6.7 million total reads per sample and an average sequence of depth of 33.7 ± 4.2 x.

### Alignment and variant calling

Fastq data was aligned against the GRCh38 human reference assembly using the BWA Burrow-Wheeler Aligner. Picard tools was used for marking duplicates (https://github.com/broadinstitute/picard) BAM les were generated using SAMtools. (http://samtools.sourceforge.net/). GATK v4.0.4.0; https://www.broadinstitute.org/gatk/) was used for base quality score recalibration, variant calling and variant quality score recalibration. Variants were annotated using VEP Variant Effect Predictor(https://genomebiology.biomedcentral.com/articles/10.1186/s13059-016-0974-4). cDNA and protein sequence variants are described in accordance with the recommendations of Human Genome Variation Society. All LRRK2 variants were harmonized with the canonical Ensemble feature ENSG00000188906; ENST00000298910 (RefSeq NM_198578.4) of the GRCh38/hg38 human reference genome build.

### Variant ltering

Following alignment, variants were filtered using specific thresholds for several annotations using the custom in-house pipeline. Synonymous and common variants with a population allele frequency (AF) ≥ 0.05 (5%) reported in the Genome Aggregation Database v3 (gnomAD v3) or Exome Aggregation Consortium (ExAC) database or reported to be common in the in-house WES database as well as variants predicted to be of functionality ‘low impact’ were removed. Missense variants were predicted *in silico* using Sort Intolerant from Tolerant (SIFT) and Polymorphism Phenotyping v2 (PolyPhen2); those labelled to be ‘benign’ or ‘tolerated’ were excluded. Additionally, the Combined Annotation Dependent Depletion (CADD) score of 20 was used as cut-off for filtering missense variants. Lastly, only variants within PD-associated genes based on Genomics England Parkinson Disease and Complex Parkinsonism panel list v1.120 (https://panelapp.genomicsengland.co.uk/panels/39/) and published literature [^75^] were included (Suppl. Table 3).

### Variant validation

Sanger sequencing was performed to confirm the variant in all positive cases. Primers for mutation validation were designed using Primer3 software [^76^]. Reference sequences of the human genome assembly build 38 (GRCh38) were obtained from the Ensemble Genome Browser (http://www.ensembl.org/index.html). The Primer-BLAST online tool (https://www.ncbi.nlm.nih.gov/tools/primer-blast/) was used to ensure the designed primers were specific to the target of interest and the designed primers ordered (Sigma-Aldrich, USA) are stated in Suppl. table 4. The PCR reaction was then optimised and confirmed through agarose gel electrophoresis (Suppl. table 3). The purified PCR product (Exosap, Thermo Fisher Scienti c, USA) were then sequenced and read by the ABI 3730xl DNA analyser (Applied Biosystems, USA). The sequencing reads were checked using Sequencer software version 4.1.4.

### Beta-glucocerebrosidase activity

Dry blood spot (DBS) samples (n = 2, filter cards CentoCard^®^), were analysed in Centogene GmbH, Rostock, Germany and the beta-glucocerebrosidase activity was measured using fluorometric assay with the reference value as ≥ 4.1μmol/L/h.

#### Structural modeling of LRRK2 L1795F missense variant

Structural image for LRRK2 dimeric complex derived from PDB 7LHT [^18^]. Molecular graphics and analyses performed with UCSF ChimeraX, developed by the Resource for Biocomputing, Visualization, and Informatics at the University of California, San Francisco, with support from National Institutes of Health R01-GM129325 and the Office of Cyber Infrastructure and Computational Biology, National Institute of Allergy and Infectious Diseases [^19^].

### Statistical analysis

Statistical analysis was performed using R version 4.3.1 (2023–06-16) and R studio (version 2023.09.1 + 494; RStudio, Inc). Demographic data were summarised descriptively using frequency counts and percentages of the total study population for categorical variables.

## Figures and Tables

**Figure 3:**
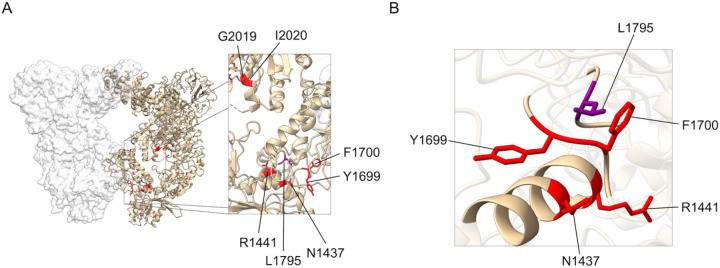


**Figure 4:** (A) CryoEM structure for the LRRK2 dimer with highlighted PD-associated mutations including the proposed p.L1795F variant (B) proximity of p.L1795F to previously demonstrated pathogenic variants in the ROC and COR domains. Image derived from PDB 7LHT using chimera X [^18^,^19^].

**Table 1:** Demographics and clinical characteristics of *LRRK2* p.L1795F positive PD patients.

Patient no.	Sex	Age (y)	Origin	Age at onset (y)	Family history of PD	Family members affected with PD
**P1**	F	48	Hungarian	25	Positive	brother, maternal aunt and grandmother
**P2**	F	55	Slovak	45	Negative	None
**P3**	M	83	Slovak	55	Positive	sister, mother, maternal grandmother
**P4**	F	80	Slovak	69	Positive	mother, mother’s sister

PD = Parkinson’s Disease; y = years; F = female; M = male

**Table 2:** Detailed phenotype of identified *LRRK2* p.L1795F positive PD patients.

*Patient no*.	*P1*	*P2*	*P3*	*P4*
**Age at onset (y)**	25	45	55	69
**Disease duration (y)**	23	10	28	11
**PD subtype**	Akinetic-Rigid	Akinetic-Rigid	Akinetic-Rigid	Akinetic-Rigid
**Initial motor features**	Unilateral bradykinesia and rigidity	Unilateral bradykinesia and rigidity	Unilateral bradykinesia and rigidity	Unilateral bradykinesia and rigidity
**MDS-UPDRS part III ON/OFF**	4 ON 48 OFF	14 ON 31 OFF	58 ON NA	29 ON NA
**Bradykinesia**	+	+	+	+
**Rigidity**	+	+	+	+
**Resting tremor**	−	−	−	−
**Freezing**	+	−	+	−
**Postural instability**	+	+	+	+
**Dyskinesia**	+	+	+	+
**H&Y stage**	3	3	5	3
**Early motor fluctuations**	+	+	+	+
**MoCA score**	29	26	13	23
**Neuropsychiatric features (self-reported)**	Depression	Depression, anxiety, apathy	None	Depression, anxiety
**Non-motor features**	fatigue, nocturia, constipation, light headedness on standing	fatigue, insomnia, urinary urgency, constipation, light headedness on standing, excessive sweating, chronic pain	fatigue, nocturia, urinary urgency, light headedness on standing, chronic pain	fatigue, insomnia, urinary urgency, light headedness on standing, chronic pain
**Other features**	Hypercholesterolemia, Endometriosis, Hydronephrosis caused by ureteric stones	Hypothyroidism	Osteoarthrosis	Arterial hypertension, Hypercholestero-lemia, Osteoarthrosis
**Response to levodopa**	+	+	+	+
**Current medication**	DBS STN, L-dopa/Carbidopa/entacapone (300/75/1200 mg/day) Pramipexole (1.04 mg/day) Amantadine (300 mg/day)	DBS STN, L-dopa/Carbidopa (250/25mg/day), Amantadin (100mg/day) Rasagiline (1mg/day) Opicapone (50mg/day)	LCIG (6,7ml/hod), Amantadin (300mg/day), Opicapone (50mg/day)	LCIG (6,7ml/hod), L-dopa/carbidopa (100/25mg/day)
**Therapeutical effect on motor fluctuations**	unsatisfactory	unsatisfactory	unsatisfactory	unsatisfactory
**GCase activity**	NA	4.9 μmol/L/h (normal)	6.0 μmol/L/h (normal)	NA

PD = Parkinson’s Disease; MDS-UPDRS – Movement Disorder Society-Unified Parkinsons Disease Scale; H&Y = Hoehn and Yahr; MoCA – Montreal Cognitive Assessment; GCase = Beta-glucocerebrosidase; L-dopa = Levodopa; LCIG = Levodopa-Carbidopa Intestinal Gel; DBS = Deep Brain Stimulation; STN = Subthalamic Nucleus; y = years; NA = not available;

## Data Availability

The data supporting this study’s findings are available from the corresponding author, upon reasonable request.
